# Fluoroquinolone Prophylaxis Uncovers High Prevalence Rates of Fluoroquinolone-Resistant *Enterobacterales* Colonization in Multiple Myeloma Autologous Transplant Patients: A Prospective Cohort Study

**DOI:** 10.3390/cancers18101566

**Published:** 2026-05-12

**Authors:** Chintan Patel, Austin J. Terlecky, Melissa Baker, Tara Lozy, Kelly K. Yen, Navjot Kaur, Lauren Machere, Alaa Ali, Christina Cho, Michele L. Donato, Pashna N. Munshi, Barry N. Kreiswirth, Scott D. Rowley

**Affiliations:** 1 John Theurer Cancer Center, 92 Second Street, Hackensack, NJ 07601, USA; chintan.patel@hmhn.org (C.P.); melissa.baker@hmhn.org (M.B.); navjotg.kaur@hmhn.org (N.K.); lauren.machere@hmhn.org (L.M.); christina.cho@hmhn.org (C.C.); michele.donato@hmhn.org (M.L.D.); 2 Center for Discovery and Innovation, 111 Ideation Road, Nutley, NJ 07110, USA; austin.terlecky@hmh-cdi.org (A.J.T.); tara.lozy@hmhn.org (T.L.); kelly.yen@hmhn.org (K.K.Y.); barry.kreiswirth@hmh-cdi.org (B.N.K.); 3 Lombardi Comprehensive Cancer Center, 3800 Reservoir Road NW, Washington, DC 20007, USA; alaa.ali@moffitt.org (A.A.); pashna.munshi@pennmedicine.upenn.edu (P.N.M.)

**Keywords:** fluoroquinolone resistance, *Enterobacterales*, antibiotic prophylaxis, autologous stem cell transplantation, multiple myeloma

## Abstract

**Background:** Fluoroquinolone prophylaxis during autologous stem cell transplantation (aSCT) reduces the risk of fever but raises the risk of bloodstream infection (BSI) with fluoroquinolone-resistant *Enterobacterales* (FRE). We performed a prospective cohort study to detect the presence and potential gain or loss of colonic FRE colonization using serial sampling before and after aSCT in a uniform population of patients with a diagnosis of multiple myeloma. **Methods:** Eligible subjects underwent aSCT after conditioning with dose-intense melphalan, 200 mg/m^2^. Peri-anal swabs were obtained before aSCT, upon hospital discharge, and 12–16 weeks after transplantation. Samples were cultured in tryptic soy broth supplemented with either ciprofloxacin or ceftriaxone with subsequent plating onto selective chromogenic agar designed to facilitate recovery and differentiation of *Enterobacterales*. **Results:** FRE colonization on pre-transplant sampling was detected for 23 of 117 subjects (19.7%) and 29 of 98 (29.6%) subjects at hospital discharge after a course of fluoroquinolone (116/117 subjects) prophylaxis (*p* < 0.001) and 28 of 92 (30.4%) subjects at 12–16 weeks. Including all three sampling time points, 48 of 117 subjects (41.0%) tested positive for FRE colonization. In total, 58 of the 90 FRE isolates (64.4%) from 48 subjects expressed extended-spectrum beta-lactamase (ESBL). Three FRE-colonized subjects developed FRE BSI. Bloodstream isolates for two subjects were identical to the organisms identified on pre-transplant sampling. **Conclusions:** We hypothesize that fluoroquinolone prophylaxis of subjects with undetected low levels of FRE colonization allows the expansion of the FRE population, placing subjects at risk of BSI with fluoroquinolone-resistant (and ESBL-expressing) *Enterobacterales*. Pre-transplant testing for FRE colonization permits patient-specific design of prophylactic and empiric antibiotic regimens.

## Simple Summary

Fluoroquinolone prophylaxis for patients undergoing autologous stem cell transplantation (aSCT) reduces the risk of fever but raises the risk of bloodstream infection (BSI) with fluoroquinolone-resistant *Enterobacterales* (FRE). We performed a prospective cohort study of colonic FRE colonization in a defined population of patients undergoing aSCT, exploring the detection of colonization with serial sampling before and after transplantation. FRE colonization on pre-transplant sampling was detected for 23 of 117 subjects (19.7%). Detection of FRE colonization doubled when including all three (pre- and post-transplant) sampling time points, and 48 of 117 subjects (41.0%) tested positive. Furthermore, 58 of the 90 FRE isolates (64.4%) from 48 subjects expressed extended-spectrum beta-lactamase (ESBL). We hypothesize that fluoroquinolone prophylaxis of subjects colonized with low levels of FRE allows the expansion of the FRE population, placing subjects at risk of BSI with fluoroquinolone-resistant (and ESBL-expressing) *Enterobacterales*. Accordingly, screening for FRE colonization permits adjustments in prophylaxis and empiric antibiotic regimens, possibly reducing serious BSI risk.

## 1. Introduction

Dose-intense melphalan chemotherapy with autologous peripheral blood stem cell (aSCT) transplantation in the treatment of multiple myeloma (MM), although well-tolerated with a treatment-related mortality risk usually less than 2% [1], incurs an obligate period of pancytopenia and chemotherapy-induced mucosal damage. Serious bloodstream infections (BSIs) may occur during this period of neutropenia, with the risk compounded by the gut mucosal damage incurred by dose-intensive chemotherapy, possibly affecting the absorption of oral antibiotics [2], and allowing transmigration of enteric organisms [3,4,5,6,7,8,9].

The recognition of this risk of opportunistic infections led to multiple studies exploring the role of prophylactic antibiotics given during the period of neutropenia [10]. A Cochran review of the use of antibiotic prophylaxis for patients at risk of neutropenia identified 109 trials involving 13,579 patients conducted between 1973 and 2010, and concluded that antibiotic prophylaxis (compared to placebo) decreased the risk of febrile neutropenia (FN), and, in some studies, the overall mortality risk [11]. Various studies demonstrated the particular efficacy and lower toxicity of fluoroquinolone (FQ) antibiotics, which are now the standard of care for various autologous or allogeneic stem cell transplants and other marrow-toxic regimens [10,12,13,14].

However, the wide-spread use of FQ has resulted in increasing incidence of colonization of the gut microbiome by fluoroquinolone-resistant *Enterobacterales* (FRE), which can be detected by pre-transplant peri-anal swab cultures [15,16]. The reported incidence of FRE colonization differs by the population being studied, with varying incidences found in different countries. Satlin et al., for example, reported a 21% incidence of FRE colonic colonization in patients undergoing autologous transplantation between 2016 and 2019 at a single center in New York [16]; Zavrelova reported a 37% incidence in their study from the Czech Republic [17]. A systemic review of antibiotic resistance patterns similarly found wide differences in antibiotic-resistant BSIs across several European countries [18]. Retrospective reviews of patients undergoing aSCT with or without FQ prophylaxis reported that FQ prophylaxis reduced the risk of BSI in patients undergoing aSCT but increased the incidence of resistant organisms [19,20,21]. aSCT recipients who are colonized with FRE pre-transplant and receive FQ prophylaxis have high rates of bacteremia from their colonizing strain [22], leading to the hypothesis that patients known to be colonized by FRE should not receive FQ prophylaxis [15,16,23,24].

Most of these studies combined subjects of various diagnoses undergoing autologous or allogeneic transplantation or leukemia remission-induction therapy. We conducted a prospective study of the incidence of FRE colonization in a uniform population of patients undergoing autologous aSCT after conditioning with dose-intense melphalan chemotherapy in the treatment of MM. The intent of the study is to determine the incidence of FRE colonization in this restricted population, to determine the gain or clearance of FRE on serial sampling at two additional times during initial recovery after transplantation, and to explore the relationship of FRE colonization with BSI and the specific toxicities of aSCT.

## 2. Methods

### 2.1. Subject Eligibility

Eligibility for this prospective study included a diagnosis of MM undergoing aSCT at one of two affiliated transplant sites (Hackensack University Medical Center (HUMC), Hackensack, NJ, or MedStar Georgetown University Hospital (MGUH), Washington DC) after conditioning with melphalan 200 mg/m^2^ as a single infusion. Patients with other plasma cell dyscrasias, any evidence of involvement with light-chain amyloidosis, and those using a different conditioning regimen were excluded from this study. Subjects could be undergoing 1st or subsequent transplantation including salvage transplantation after a prior relapse. Only one transplant course was evaluated for each subject. Eligibility criteria also included the collection before transplantation of a peri-anal swab for analysis. Most subjects received initial remission-induction therapy outside these two institutions and a reliable history of FQ or other antibiotic exposure before aSCT could not be obtained. Per study protocol, all subjects were monitored for infectious or non-infectious complications of transplantation for 84 days (12 weeks) after transplantation.

The Institutional Review Board approved the study protocol (ClinicalTrials.gov ID NCT05824689) and all subjects provided informed consent for enrollment into the study. In total, 124 subjects enrolled in the study (105 HUMC; 19 MGUH). Seven subjects did not proceed to transplantation in the study, including two subjects who withdrew consent before transplantation, one who received an alternate conditioning regimen, two subjects who failed to collect the immediate pre-transplant specimen, and two subjects who failed to collect peripheral blood stem cells (PBSCs) or were medically ineligible to proceed to transplantation, leaving 117 subjects for evaluation (Table 1).

### 2.2. Sample Collection and Analysis

Peri-anal swabs were obtained before initial FQ prophylaxis was prescribed for the subjects undergoing chemotherapy mobilization of PBSCs (data given for reference only and not otherwise included in analysis), and for all subjects immediately before transplant admission, at time of hospital discharge, and again about day 100 (12–16 weeks) after transplantation (Table 2 and Table S1). Per protocol, all evaluable subjects collected the baseline immediate pre-transplant sample. Several subjects declined collection of the post-transplant samples (18 and 25 subjects for hospital discharge and/or day 100 time points, respectively) but, as per the protocol, remained eligible for analysis of pre-transplant FRE-colonization and subsequent transplant outcomes. Subjects were allowed to collect their own samples after instruction by study personnel.

Swab specimens were placed in Amies transport medium and immediately frozen at −70 °C to −80 °C until processing. For the detection of FQ-resistant *Enterobacterales* and extended-spectrum β-lactamase-producing *Enterobacterales* (ESBL-E), 100 μL of thawed Amies medium was inoculated into 5 mL of tryptic soy broth supplemented with either ciprofloxacin (5 ug/mL, for FRE detection) or ceftriaxone (4 ug/mL, for ESBL-E detection) per Clinical and Laboratory Standards Institute guidance for broth cultures [25]. Broths were incubated overnight at 37 °C. The following day, 100 μL aliquots from each enrichment broth were plated onto selective chromogenic agar (CHROMagar™, Saint-Denis, France) designed to facilitate recovery and differentiation of *Enterobacterales*. Strains not speciated on CHROMagar were analyzed by 16S sequence analysis. Agar diffusion plating was used to confirm phenotypic resistance for both FQ and ceftriaxone. The presence of growth confirmed antibiotic resistance [25]. Representative colonies from selective plates were subsequently archived in glycerol stocks at −80 °C for downstream analyses. No quantitative cultures were performed.

To confirm the adequacy of sample collection and bacterial viability, 100 μL of unprocessed Amies transport medium from each swab was simultaneously inoculated onto a non-selective blood agar plate and incubated for 24 h at 37 °C. The presence of growth confirmed successful collection of viable bacteria.

For subjects who subsequently developed a BSI, both the colonizing isolates and the bloodstream isolate were subjected to whole genome sequencing (WGS). Sequencing was performed using the Illumina NextSeq platform (San Diego, CA, USA) according to manufacturer protocols. Single-nucleotide variant (SNV) differences between paired colonizing and bloodstream isolates were calculated and compared against inter-patient FRE SNV distances to assess relatedness. Genomic analyses also included the identification of antimicrobial resistance determinants. Organisms isolated from urine cultures were not included in this study and were not subjected to genomic or phenotypic analyses.

### 2.3. Transplant Management and Supportive Care—PBSC Mobilization and Collection

Peripheral blood stem cells (PBSCs) were mobilized by standard procedure cytokine or chemotherapy-based regimens per transplant physician decision and were not determined by study protocol. Per institutional standard procedures, the target of CD34+ cells to be collected was at least 8–12 × 10^6^/kg to allow multiple transplants using a target dose of ≥4 × 10^6^ CD34+ cells/kg/transplant. PBSC product collection procedures were performed with a continuous flow blood cell separator. PBSC products were cryopreserved in fractions in 5.0% *w*/*v* dimethyl sulfoxide and 5% *w*/*v* human serum albumin. Products were placed in a −80 °C mechanical freezer for initial cooling and were moved the following day to a vapor-phase liquid nitrogen refrigerator for storage below −150 °C.

For cytokine mobilization, PBSCs were mobilized with granulocyte colony-stimulating factor (G-CSF) 12 µg/kg actual body weight (ABW) by daily subcutaneous injection (SC), starting 4 days before the start of apheresis. Plerixafor 0.24 mg/kg SC daily could be added, also per physician discretion. G-CSF was continued daily until the completion of the collection procedures.

PBSCs were mobilized with a chemotherapy-based regimen (CDE) consisting of single doses of dexamethasone 10 mg IV, cyclophosphamide 2 g/m^2^ IV, and etoposide 200 mg/m^2^ IV (given on day 1 of the regimen), followed by G-CSF 12 µg/kg SC daily starting on day 5. Subjects who underwent a chemotherapy mobilization regimen also received a prophylactic 5-day course of levofloxacin 500 mg daily starting on day 5 of the cycle until the resolution of neutropenia. A peri-anal swab prior to mobilization was requested for these subjects (Table 2). Leukapheresis (described above) began upon hematopoietic recovery from the debulking chemotherapy, defined as an absolute neutrophil count (ANC) of greater than 1 × 10^9^/L and a platelet count greater than 20 × 10^9^/L.

Ten subjects enrolled in this study had PBSCs remaining after a prior aSCT and the mobilization regimen is not described for these subjects.

### 2.4. Transplant Conditioning

All subjects underwent transplant conditioning with melphalan 200 mg/m^2^ (ABW) by intravenous (IV) infusion over 60 min. No adjustments in melphalan dosing for obesity or any decrease in creatinine clearance were made. Oral cryotherapy prophylaxis was encouraged during the melphalan infusion and for 60 min after the infusion completed to reduce the risk of oral mucositis. Aggressive hydration was used for uroprotection during the time of melphalan administration and for 24 h after. PBSCs were infused on the following day, at least 24 h after completion of the melphalan infusion and is defined as occurring on day +0. The dose of PBSCs was not defined by study protocol, but a target of 4 × 10^6^ CD34+ cells/kg was routinely used.

### 2.5. Hospitalization

All subjects were admitted to the inpatient transplant unit for transplantation and were discharged after achieving neutrophil engraftment, resolution of any regimen-related severe gastrointestinal (GI) toxicities, and control of any infectious complications.

### 2.6. IV Access

A dual-lumen central venous catheter (Hickman or Permcath) suitable for apheresis procedures was inserted prior to the PBSC collections for patients with limited venous access or per physician preference (74 of 91 subjects at HUMC (10 subjects had cells previously collected); 16 of 16 subjects at MGUH). If the catheter was removed before transplant admission, a dual-lumen peripherally inserted central catheter (PICC) was placed for venous access (all subjects at HUMC). The catheters were removed at the time of hospital discharge or as soon as possible after discharge.

### 2.7. Prophylactic Antibiotics

All subjects received a standard transplant antimicrobial prophylaxis regimen commencing on the day of hospital admission consisting of valacyclovir 500 mg po daily continuing through day 84, fluconazole 400 mg po daily through hospital discharge (or discontinuation of corticosteroids if administered during inpatient hospitalization), and either ciprofloxacin 500 mg po twice daily (HUMC) or levofloxacin 500 mg po daily (HUMC and MGUH) (or alternate prophylaxis for two subjects per physician discretion after study enrollment) until resolution of neutropenia. The HUMC transplant program substituted levofloxacin for ciprofloxacin prophylaxis after enrollment of participant number FRE-024. One participant (FRE-056, subject numbers shown in Supplemental Table S1) declined fluoroquinolone (FQ) prophylaxis and received a prophylaxis regimen of cefdinir, as selected by the physician caring for the participant. A second participant was admitted for transplantation receiving a course of amoxicillin/clavulanate for the management of a dental problem and was later switched to FQ.

### 2.8. Management of Neutropenic Fever

Neutropenic fever was defined as a temperature ≥ 38.0 °C in the setting of neutropenia (ANC < 0.5 × 10^9^/L). Blood cultures were obtained at the onset of fever. The prophylactic FQ (or other antibiotic) was discontinued and empiric treatment with cefepime 2 gm IV q8 h (or aztreonam 2 gm IV q8 h with vancomycin 15 mg/kg (ABW) IV q12 h for subjects with penicillin allergy) was initiated. Meropenem 1 gm IV q8 h or piperacillin–tazobactam 4.5 gm IV q6 h could be used per physician discretion. Vancomycin was added for patients with known MRSA colonization detected on pre-transplant screening. Empiric treatment continued until neutrophil engraftment and resolution of fever. Repeat blood cultures were obtained at 3-day intervals for persistent fevers or for other clinical indications. The empiric regimen could be adjusted per physician discretion in response to a documented bloodstream (BSI) or other infection, persistent fever not responsive to the antibiotic regimen, or another medical indication.

Testing for toxin-producing Clostridium difficile was performed for each participant at time of diarrhea onset, and repeat testing was performed at 3-day intervals for subjects with sustained diarrhea. Toxin-producing Clostridium difficile was treated with a course of oral vancomycin.

All subjects underwent pre-transplant testing for prior exposure to Cytomegalovirus (CMV, Table 1), hepatitis B, hepatis C, and human immunodeficiency virus (HIV), and for colonization by methicillin-resistant Staphylococcus aureus (MRSA, Table 1). No subject tested positive for hepatitis B or C, or HIV on pre-transplant screening.

Routine post-transplant surveillance testing for viral or other opportunistic infections (viral and fungal) was not prescribed in the study protocol or in the standard treatment procedures for subjects with MM undergoing aSCT at these centers.

### 2.9. Blood Component Support and Engraftment

All subjects received G-CSF 5 mcg/kg (dose rounded to vial size) every other day starting on day +3, then daily commencing on day +9 until achieving a sustained absolute neutrophil count of ≥0.5 × 10^9^/L. Thrombopoietin-mimetic agents and erythropoietin were not administered during the inpatient course.

Leukocyte-depleted and irradiated blood products were given prophylactically for a blood hemoglobin level < 7 gm/dL or a platelet count < 10 × 10^9^/L.

Neutrophil engraftment was defined as the first day after neutrophil nadir with a rising and sustained ANC of ≥0.5 × 10^9^/L. Platelet engraftment was defined as the first day after platelet nadir with a rising platelet count of ≥20 × 10^9^/L without platelet transfusion within the preceding 48 h.

### 2.10. Definition and Management of Post-Transplant Engraftment Syndrome

Subjects with persistent, non-infectious (culture-negative) fever with accompanying diarrhea consistent with “engraftment syndrome” were managed with a course of systemic corticosteroids determined by the physician caring for the participant [26,27]. Corticosteroids were prophylactically administered (per physician discretion) to 44 subjects of older age or deemed to be at higher risk of engraftment syndrome. These subjects were removed from the analysis of this endpoint. Corticosteroids were initiated (per physician discretion) for 17 subjects with non-infectious fever and diarrhea during the initial transplant course at a median 9 days after transplantation. One additional participant received corticosteroids after re-hospitalization on day 17 for non-infectious fever.

### 2.11. Post-Discharge Management

Patients were hospitalized until neutrophil engraftment, control of any infections, and resolution of severe regimen-related complications. Patients were scheduled for their first outpatient visit within 3–4 days after discharge, and were subsequently discharged to their primary oncologist. In general, patients were allowed to return to work or similar activities at 28 days after transplantation. Patients were scheduled to return to the transplant clinic for follow-up at 100 days or later after transplantation to meet transplant registry data submission requirements.

### 2.12. Statistical Analysis

The primary endpoint of this study was the prevalence of FRE colonization at the time of transplant admission, and all subjects who obtained the pre-admission sample were eligible for analysis. Secondary endpoints included the effect of FRE colonization on the risks of fever during neutropenia (FN), BSI and other infections, and on engraftment kinetics, duration of hospitalization, and the development of engraftment syndrome. We also explored the incidence of FRE colonization at the time of transplant discharge and again about 100 days after transplantation. In total, 18 and 25 subjects, respectively, (Table 2) declined collection of these two later samples but were still considered eligible for evaluation of the primary endpoint and secondary clinical endpoints occurring before study dropout. Subjects were classified for analysis as either FRE positive on the pre-transplant sample (primary analysis) or as FRE positive on any of the 3 samples (secondary analysis). Categorical variables among the FRE carriers and non-carriers are summarized using frequencies (percentages). Likewise, continuous variables are summarized using mean with standard deviation or median with interquartile range, depending on the data distribution. Comparisons of continuous variables between the FRE carriers and FRE non-carriers were performed using two-sided *t* tests or two-sided Wilcoxon rank-sum tests, as appropriate. Comparisons of categorical variables between the two groups, FRE carriers and FRE non-carriers, were conducted using Fisher’s exact test or Pearson’s Chi-square test, as appropriate. The limited number of subjects enrolled at MGUH precluded analysis of center effects and data from both centers were combined for analysis. Data were analyzed using complete cases; missing data were not imputed. In order to reduce false positives resulting from multiple hypothesis testing, a *p*-value of ≤0.01 is considered significant.

### 2.13. Role of the Funding Source

Grants and institutional funds supported this study. These sponsors were not involved in study design; in the collection, analysis, and interpretation of data; in the writing of the report; or in the decision to submit the paper for publication.

## 3. Results

### 3.1. Detection of FRE Colonization

Pre-transplant samples were collected at a median of 5 days (interquartile range (IQR): seven to three) before transplantation. Four subjects had a delay to transplantation of >28 days after sample collection for the management of medical complications per the discretion of the treating physician (data for these subjects are not excluded from analysis). FRE colonization on admission was detected for 23 of 117 subjects (19.6%, Table 2 and Table S1). Isolates were identified as *Escherichia coli* (*n* = 14), *Klebsiella* species (*n* = 6), or both (*n* = 3); 15 of the isolates (12 subjects) also demonstrated extended-spectrum beta-lactamase (ESBL) expression. The prevalence of FRE colonization was not predicted by subject characteristics such as race, sex, age, disease classification or stage, or prior treatments (Table 1). Only three of the 355 collected swabs failed quality control (QC) to demonstrate any growth on blood agar (FRE-029 (hospital discharge sample), FRE-096 and FRE-102 (day 100 samples); Supplemental Table S1). These specimens were excluded from analysis. Overall, 14 (11 and three FRE-negative and FRE-positive, respectively, at pre-transplant) out of 117 (12.0%) eligible participants did not have any post-transplant samples available for analysis after either subject refusal or failed QC.

A higher proportion of subjects tested positive for FRE (29/98, 29.6%; Table 2 and Table S1; *p* < 0.001) at hospital discharge (median discharge day 13, IQR: 12–14 days). Isolates at this time point were *Escherichia coli* (*n* = 14), *Klebsiella* species (*n* = 11), or both (*n* = 4). Notably, 15 of the subjects demonstrating FRE colonization at discharge did not test positive pre-transplant and FRE colonization was not detected on the discharge sample for six subjects who tested positive pre-transplant. A second “day 100” post-transplant (planned for 12–16 weeks post-aSCT) sample was collected for 92 subjects at 48–120 days (median, 101.5 days) with FRE colonization found for 28 of 90 evaluable subjects (31.1%, two samples that failed QC steps are excluded from this analysis). Isolates were identified as *Escherichia coli* (*n* = 14), *Klebsiella* species (*n* = 11), or both (*n* = 3). Four of the six subjects testing positive on the pre-transplant sample but negative on the discharge specimen tested positive on this third sample (Supplemental Table S1). Including all three time points, a total of 48 of 117 subjects (41.0%) tested positive for FRE colonization and 58 of the 90 FRE isolates (64.4%) identified from 48 subjects also expressed ESBL.

### 3.2. Post-Transplant Infectious Complications

Fevers (>38.3 °C) occurred in 28 subjects during the period of neutropenia (Table 3). Pre-transplant colonization by FRE did not predict fever or time of fever onset during the period of neutropenia.

*Enterobacterales* species were detected in the blood cultures of three subjects (all FRE colonized, Table 3 and Table 4) on pre-transplant samples and *Streptococcus mitis* in one subject (Table 3). One subject (FRE-056) undergoing salvage transplant was the only subject who did not receive FQ prophylaxis during the transplant hospitalization. The *Enterobacterales* for these three subjects were FQ resistant or of intermediate sensitivity. None of any follow-up blood cultures or blood cultures obtained on re-admission showed bacterial growth. Pre-transplant colonization by FRE predicted the development of *Enterobacterales* BSI (Table 4, *p* = 0.006).

A comparison of WGS performed on isolates from both the blood and stool confirmed the identity of two of the three subjects (Table 5). One additional subject developed an FQ-resistant ESBL *Escherichia coli* urinary tract infection (UTI) without a BSI. Diarrhea prompting evaluation and treatment occurred in 86 subjects, but only three subjects (Table 3) of the 86 tested positive for toxin-producing *Clostridium difficile* (one subject FRE colonized on admission and a second FRE colonized at a later testing point).

The only other infections occurring during the initial hospital stay were symptomatic respiratory viral infections in three subjects, with nasal swab PCR panel testing returning positive for rhinovirus (one subject, FRE-073, day 2) and SARS-CoV-2 (two subjects, FRE-105 and MFRE-015, days 4 and 5, respectively). Only one of these three subjects tested positive for FRE colonization on admission (FRE-105); one subject tested positive for FRE colonization only on the day 100 sample (FRE-073). These infections did not result in a prolonged initial hospital stay, with all three subjects discharged home on day 11 (one subject) or 12 (two subjects).

### 3.3. Hematological Recovery, Duration of Hospitalization, and Engraftment Syndrome

A total of 116 subjects achieved neutrophil and platelet engraftments at medians of 11 and 13 days after transplantation, respectively (Table 4, an accurate engraftment date for one subject was not obtained). The median duration of transplant hospitalization after transplantation was 13 days for both groups and no deaths occurred during the transplant course or study-defined follow-up period. The presence of FRE colonization at the time of transplant admission did not predict absolute neutrophil count (ANC), platelet engraftment kinetics, requirements for blood component transfusions, or duration of hospitalization. Analysis of engraftment syndrome was limited to the subgroup of subjects who did not receive prophylactic corticosteroids. Engraftment syndrome was not predicted by FRE colonization.

### 3.4. Post-Transplant Re-Admission

Fourteen subjects (Table 6) were re-hospitalized within 12 weeks after transplantation on 17 occasions for fever or infection (12 events) or for non-infectious reasons (five events). Two subjects had a second post-transplant re-admission for the management of SARS-CoV-2 infection and one subject had a third re-admission for fever. Re-hospitalization occurred for 7.4% of the non-FRE-colonized subjects (initial FRE testing) but 30.4% of the FRE-colonized subjects (Table 4). Detection of FRE colonization on pre-transplant testing predicted for subsequent re-hospitalization (*p* = 0.002); the detection of FRE colonization, including the post-transplant samples, was of borderline significance (*p* = 0.06). A causal relationship between fever and FRE colonization could not be determined. Five of the seven subjects who presented with a diagnosis of fever not otherwise explained, prompting re-hospitalization, were found to be FRE colonized at the pre-admission or discharge time points compared to three of the five subjects hospitalized for non-infectious reasons. Blood cultures for the seven subjects with fever on re-admission did not detect BSI.

## 4. Discussion

Peri-anal swabs are described to be a reliable specimen type for detection of colonic colonization with FRE, with studies showing a 95% concordance with stool cultures [28,29]. The 19.6% prevalence of FRE colonization detected immediately before transplant in our population is consistent with the reports from other United States transplant centers obtaining samples at a single pre-transplant time point in mixed populations of patients undergoing autologous or allogeneic transplantation [16].

In contrast to most other reports, we performed and reported serial testing at two time points after the transplant process. The 41.0% prevalence we found when all sample points for each subject were combined is markedly concerning, especially because 64.4% of the isolates also expressed ESBL (including 34 of the 38 FQ-resistant *Klebsiella* sp. isolates). This difference in the detection of FRE colonization (and the occasional FRE-negative test after a prior positive) on serial testing could result from low test sensitivity, although this hypothesis is not supported by the close correlation of peri-rectal swabs and stool cultures reported by others [28,29] and the low failure rate of sample cultures (three of 355 samples submitted for analysis). We did not attempt to schedule sampling relative to the timing of skin hygiene and allowed patient-collected sampling, which may have contributed to the variation found in serial testing for some of the subjects. Although also conceivable, we do not believe the increase in FRE colonization detected on post-transplant sampling resulted from nosocomial exposure to FRE based on the strict isolation practices maintained in the dedicated transplant units during inpatient hospitalization.

Rather, we hypothesize that the 41.0% positive rate with serial sampling more accurately describes the true prevalence of FRE colonization in this population, and this higher detection rate results from an expansion in the FRE population in the gut microbiome after a course of FQ prophylaxis such that detection of a now-dominant FRE population becomes more likely [30,31,32]. For this study we used a cell enrichment technique consisting of the growth of viable bacteria in selective (antibiotic-containing) broth media, allowing the growth of bacterial populations up to a detectable level. This is considered the “gold-standard” method by which other detection techniques are to be compared [33,34]. That these broth cultures did not detect a small FRE-colonizing population until after the initial transplant procedures may reflect the poorly understood interplay between endogenous factors such as the microbiome, colonic mucosal characteristics, and the pathogen being studied, and exogenous factors including the administration of antibiotics [35]. It is also conceivable that, under pressure from the prophylactic antibiotics, changes in resistance gene(s) expression occurred, resulting in increased fluoroquinolone resistance of the *Enterobacterales* and thus becoming detectable in the enrichment broth cultures used in this study [36]. Specific studies of the resistance patterns of colonic *Enterobacterales* were not a feature of this study and would be required to determine if our subjects developed increasing resistance under antibiotic pressure versus a flux in the microbiome, allowing expansion of an existing but minute FRE population.

Others reported that longitudinal studies of the intestinal microbiome correlated with even small increases in bacterial dominance with subsequent BSI with the same organisms [30,31]. Three of the four bacterial BSI were FRE, two of which were identical to the colonizing species. We can only speculate if additional FRE BSIs, particularly in subjects with post-transplant FRE colonization, would have occurred if the period of neutropenia was longer. That only three subjects developed a BSI during the initial transplant course in this study likely reflects our choice to restrict enrollment to subjects with myeloma undergoing autologous aSCT, the limited toxicity of the transplant conditioning regimen, and the expected prompt resolution of neutropenia and regimen-related mucositis in this patient population. Studies of FRE in patients undergoing allogeneic transplantation report a much higher incidence of BSI, ranging from 26 to 43% [16,22]. In those studies, most of the BSIs in FRE-colonized patients were caused by the colonizing strains of FRE, also observed in this study. A relationship between colonic colonization and BSI is also described in studies of various antibiotic-resistant BSIs [37,38,39]. Accordingly, the low risk of BSI found in our study cannot be translated to patient populations receiving more intensive or immunosuppressive regimens, or experiencing longer periods of neutropenia or greater degrees of mucositis.

None of the subject characteristics we examined predicted FRE colonization. Furthermore, we did not find that FRE colonization predicted most of the various transplant outcomes examined, such as the duration of neutropenia or thrombocytopenia or the duration of hospitalization. FRE colonization predicted for re-admission after initial hospital discharge for the management of low-grade fever but without BSI detected. An explanation for this correlation between FRE colonization and post-transplant re-admission is not obvious. Non-infectious diarrhea consistent with engraftment syndrome was common, with 53% of subjects receiving short courses of corticosteroids given either prophylactically or in response to GI toxicity with non-infectious (culture-negative) fever, but not predicted by FRE colonization. Diarrhea prompted testing for *Clostridium difficile* toxin in 86 subjects but only three tested positive for this infection. This small number of subjects precludes determining a relationship between FRE and *Clostridium difficile* colonizations.

We are unable to determine if FRE colonization is a population-wide consequence of wide-spread FQ use or if our subject population was more likely to be exposed to FRE compared to the healthy population in these metropolitan areas [40]. We cannot determine from our data the timing or source of colonization by FRE without access to a reliable history of prior FQ or other antibiotic exposure or a history of exposure to populations with a high prevalence of FRE colonization through travel, nor can we comment on the natural history of FRE colonization, including if FRE colonization is maintained or lost over a longer time period then followed in this study.

Although we report a low risk of BSI in our subjects with FRE colonization, the adoption of routine detection of FRE colonization by analysis of peri-anal swabs may contribute to a more patient-specific empiric antibiotic regimen for neutropenic fever by predicting the antibiotic sensitivity of possible BSIs. We also emphasize that 64.4% of the FRE were also ESBL-producing organisms, which is especially relevant for transplant programs that use a cephalosporin as a first choice in treating neutropenic fever. We agree with the recommendation by Satlin et al. and others that, in consideration of the current risk of antibiotic-resistant colonization in the various patient populations, pre-treatment screening leading to patient-specific prophylaxis regimens may be adopted, rather than uniform application of prophylaxis and empiric regimens [16,38]. An undetected FRE population may become evident after exposure to FQ antibiotics and serial testing in the management of the neutropenic patient may be beneficial in appropriate clinical settings. This also warrants continued exploration of more sensitive methods to detect low levels of FRE [33,34], and even the study of possible decolonization techniques [40]. Centers with a high incidence of drug-resistant *Enterobacterales* should consider avoidance of routine antibiotic prophylaxis for select patient populations at lower risk of serious BSI, possibly reducing the risk of BSI with multi-drug-resistant organisms.

## 5. Conclusions

In this prospective cohort study, FRE colonization was detected in a not-insignificant minority of subjects undergoing aSCT. The risk of BSI with FRE in this restricted study population of subjects undergoing treatment of MM was low, likely related to the brief period of neutropenia and modest gastrointestinal toxicity of the melphalan conditioning regimen. After a routine course of FQ prophylaxis, the detection of FRE colonization doubled (from 19.6% pre-transplant to 41.0% all time points combined). Our analysis suggests that a population of the subjects who tested negative for FRE colonization before transplantation were colonized with undetected low levels of FRE and that FQ prophylaxis allowed the expansion to a now-dominant colonic FRE population, placing these subjects at risk of BSI with FRE. An alternate explanation is the possibility of a prophylaxis-related pressure on FQ-resistant gene expression. Furthermore, the majority of FRE isolates (58/90, 64.4%) also expressed ESBL. Other patient populations such as those undergoing allogeneic stem cell transplantation or leukemia remission-induction therapy face higher risks of BSI compared to our study population, and studies in those groups also show correlations between gut colonization by antibiotic-resistant organisms and BSI with the same organisms. We propose that the adoption of routine pre-treatment screening for FRE colonization, leading to patient-specific prophylaxis and empiric antibiotic regimens rather than uniform application of antibiotic regimens, should be considered for the various patient populations undergoing autologous or allogeneic transplantation.

## Figures and Tables

**Table 1:** Subject Demographics classified by FRE colonization detected on pre-transplant sample only or including post-transplant samples.

Subject Demographics	Pre-Transplant Sample			Any of the Three Time Points Sampled		
FRE Colonization	Negative *n* = 94	Positive *n* = 23	*p* Value	Negative *n* = 69	Positive *n* = 48	*p* Value
Transplant site ^a^			0.92			0.16
HUMC	81 (86.2%)	20 (87.0%)		57 (82.6%)	44 (91.7%)	
MGUH	13 (13.8%)	3 (13.0%)		12 (17.4%)	4 (8.3%)	
Transplant number ^a^			0.11			0.20
1	88 (93.6%)	19 (82.6%)		65 (94.2%)	42 (87.5%)	
>1	6 (6.4%)	4 (17.4%)		4 (5.8%)	6 (12.5%)	
Sex ^a^			0.86			0.47
M	55 (58.5%)	13 (56.5%)		42 (60.9%)	26 (54.2%)	
F	39 (41.5%)	10 (43.5%)		27 (39.1%)	22 (45.9%)	
Race *^,a^			0.53			1.00
White	56 (74.7%)	14 (70%)		44 (73.3%)	26 (74.3%)	
Black	18 (24%)	5 (25%)		15 (25.0%)	8 (22.9%)	
Other	1 (1.3%)	1 (5%)		1 (1.7%)	1 (2.9%)	
Age at transplant ^b^	64.4	65.1	0.68	64.8	64.5	0.59
	(55.4–72.7)	(58.2–71.4)		(55.5–72.8)	(57.2–71.1)	
BSA (m^2^) ^c^	2.0 (0.3)	1.9 (0.3)	0.73	2.0 (0.2)	2.0 (0.3)	0.89
Myeloma subclass ^a^			0.12			0.37
IgG	61 (64.9%)	14 (60.9%)		46 (66.7%)	29 (60.4%)	
IgA	20 (21.3%)	2 (8.7%)		14 (20.3%)	8 (16.7%)	
Light Chain Disease	13 (13.8%)	7 (30.4%)		9 (13.0%)	11 (22.9%)	
Myeloma light chain subclass ^a^			0.22			0.96
Kappa	73 (77.7%)	15 (65.2%)		52 (75.4%)	36 (75.0%)	
Lambda	21 (22.3%)	8 (34.8%)		17 (24.6%)	12 (25.0%)	
R-ISS stage at diagnosis *^,a^			0.27			0.02
I	23 (37.7%)	4 (26.7%)		21 (44.7%)	6 (20.7%)	
II	27 (44.3%)	10 (66.7%)		17 (36.2%)	20 (69.0%)	
III	11 (18.0%)	1 (6.7%)		9 (19.2%)	3 (10.3%)	
No. cycles induction therapy *^,b^	4 (3–5)	4 (3–4)	0.64	4 (3–5)	4 (3–5)	0.80
Prior daratumumab use ^a^			0.89			0.20
Yes	30 (31.9%)	7 (30.4%)		25 (36.2%)	12 (25.0%)	
No	64 (68.1%)	16 (69.6%)		44 (63.8%)	36 (75.0%)	
PBSC mobilization ^a^			0.19			0.37
CDE	40 (42.6%)	7 (30.4%)		27 (39.1%)	20 (41.7%)	
G-CSF/plerixafor	48 (51.1%)	12 (52.2%)		38 (55.1%)	22 (45.8%)	
Prior stored cells	6 (6.4%)	4 (17.4%)		4 (5.8%)	6 (12.5%)	
Apheresis catheter placed *^,a^			0.99			0.07
Yes	74 (84.1%)	16 (84.2%)		58 (89.2%)	32 (76.2%)	
No	14 (15.9%)	3 (15.8%)		7 (10.8%)	10 (23.8%)	
Co-morbidity score *^,a^			0.71			0.84
0–3	50 (62.5%)	11 (57.9%)		35 (62.5%)	26 (60.5%)	
4–10	30 (37.5%)	8 (42.1%)		21 (37.5%)	17 (39.5%)	
Cytomegalovirus status ^a^			0.71			0.87
Pos	49 (52.1%)	13 (56.5%)		37 (53.6%)	25 (52.1%)	
Neg	45 (47.9%)	10 (43.5%)		32 (46.4%)	23 (47.9%)	
Creatinine ^b^	0.80	0.88	0.22	0.80	0.83	0.23
	(0.70–0.91)	(0.72–1.09)		(0.70–0.89)	(0.70–1.05)	
Albumin ^b^	4.0	4.1	0.80	4.0	4.1	0.86
	(3.9–4.2)	(3.9–4.2)		(3.9–4.2)	(3.8–4.2)	
Diabetes ^a^			0.21			0.18
Yes	11 (11.7%)	5 (21.7%)		7 (10.1%)	9 (18.8%)	
No	83 (88.3%)	18 (78.3%)		62 (89.9%)	39 (81.3%)	
MRSA colonized *^,a^			0.99			0.95
Yes	4 (4.4%)	1 (4.3%)		3 (4.5%)	2 (4.3%)	
No	87 (95.6%)	22 (95.7%)		64 (95.5%)	45 (95.7%)	

Shown are clinical details for subjects enrolled in the study and eligible for analysis, classified by FRE colonization at time of pre-transplantation samples (primary analysis) or FRE colonization including the two post-transplant samples (secondary analysis). FRE = fluoroquinolone-resistant *Enterobacterales*, HUMC = Hackensack University Medical Center, MGUH = Medstar Georgetown University Hospital, BSA = body surface area, R-ISS = Revised International Staging System, PBSC = peripheral blood stem cell, CDE = cyclophosphamide, etoposide, and dexamethasone, G-CSF = granulocyte colony-stimulating factor, MRSA = methicillin-resistant *Staphylococcus aureus*. ^a,b,c^ Data are shown as (a) n (%), (b) median (IQR) or (c) mean (SD). See Methods for a description of statistical analysis. * Data not available for all subjects.

**Table 2:** Incidence of FRE colonization.

Sample Point	No of Subjects	FRE Pos *	FRE and ESBL Pos Isolates ^+^
Pre-CDE mobilization	47	11 (23.4%)	4 (36.3%)
Pre-transplant	117	23 (19.6%)	15 (57.7%)
Hosp discharge	98 ^#^	29 (29.6%)	21 (63.6%)
Day 100 (12–16 weeks)	90 ^#^	28 (31.1%)	22 (71.0%)

* Shown are the number of subjects positive for FRE colonization by time point of testing. CDE = cyclophosphamide, etoposide, and dexamethasone, FRE = fluoroquinolone-resistant *Enterobacterales*, ESBL = extended-spectrum beta-lactamase. ^+^ Shown are the number (and proportion) of the *Enterobacterales* isolates that expressed extended-spectrum beta-lactamase. Note, two isolates may have been detected for some participants. Individual participant and isolate results are shown in Supplemental Table S1. ^#^ Samples for one subject at discharge and two subjects at day 100 failed quality control steps and are excluded from this analysis. Data are shown as n (%). See Methods for a description of statistical analysis.

**Table 3:** Documented bacterial infections during initial hospital stay.

Subject Pre-Transplant Sample	FRE Neg	FRE Pos	*p* Value
Febrile neutropenia ^a^			0.79
Yes	22 (23.4%)	6 (26.1%)	
No	72 (76.6%)	17 (73.9%)	
Time of fever onset (Days) ^b^	8 (6–9)	7 (4.25–8.25)	0.35
*Enterobacterales* BSI			ND
FRE/ESBL-positive organism ^+^	0	1	
FRE-positive organism	0	2	
Urinary tract infection	0	1	ND
Toxigenic *Clostridium difficile* detection	2	1	ND
Other BSI (*Streptococcus mitis*)	1	0	ND

Shown are incidence of fever, and incidence of BSI, urinary tract infection (UTI), and other infections during initial hospital stay for subjects classified by FRE colonization on pre-transplant sample. FRE = fluoroquinolone-resistant *Enterobacterales*, BSI = bloodstream infection, ESBL = extended-spectrum beta-lactamase, ND = not done. ^+^ All 3 organisms identified in blood cultures were fluoroquinolone resistant and 1 also expressed ESBL production. ^a,b^ Data are shown as (a) n (%) or (b) median (IQR). See Methods for a description of statistical analysis.

**Table 4:** Transplant details for subjects classified by FRE colonization detected on pre-transplant sample only or including post-transplant samples.

Transplant Factors	Pre-Transplant Sample			Any of the Three Time Points Sampled		
FRE Colonization	Negative *n* = 94	Positive *n* = 23	*p* Value	Negative *n* = 69	Positive *n* = 48	*p* Value
Melphalan Dose (mg, total) ^c^	392.6 (51.5)	387.7 (50.3)	0.68	391.2 (48.8)	392.4 (54.6)	0.90
PBSC CD34+ dose	4.76	4.78	0.66	4.79	4.73	0.81
(×10^6^/kg actual body weight) ^b^	(4.22–5.32)	(4.08–5.84)		(4.23–5.32)	(4.15–5.71)	
Engraftment (Days) ^b^						
ANC	11 (10–11)	11 (11–11)	0.23	11 (10–11)	11 (11–11)	0.32
Platelet	13 (11–14)	13 (11–15)	0.53	13 (11–14)	13 (11–15)	0.12
Transfusions ^b^						
RBC units	0 (0–0)	0 (0–0)	0.61	0 (0–0)	0 (0–1)	0.10
Platelet products	1 (0–1)	1 (0–2)	0.05	1 (0–1)	1 (0–2)	0.07
Antibiotic prophylaxis ^#,a^			0.47			0.12
Ciprofloxacin	19 (20.43%)	3 (13.64%)		10 (14.49%)	12 (26.09%)	
Levofloxacin	74 (79.57%)	19 (86.36%)		59 (85.51%)	34 (73.91%)	
Febrile neutropenia ^a^			0.79			0.82
Yes	22 (23.4%)	6 (26.1%)		16 (23.2%)	12 (25.0%)	
No	72 (76.6%)	17 (73.9%)		53 (76.8%)	36 (75.0%)	
*Enterobacterales* positive blood culture *^,a^			0.006			0.07
Yes	0 (0.0%)	3 (50.0%)		0 (0.0%)	3 (25.0%)	
No	22 (100.0%)	3 (50.0%)		16 (100.0%)	9 (75.0%)	
ICU transfer ^a^			0.62			0.40
Yes	1 (1.1%)	0 (0.0%)		1 (1.5%)	0 (0.0%)	
No	93 (98.9%)	23 (100.0%)		68 (98.5%)	48 (100.0%)	
Unplanned steroid administration ^a^			0.34			0.65
Yes	14 (31.1%)	3 (18.8%)		10 (30.3%)	7 (25.0%)	
No	31 (68.9%)	13 (81.2%)		23 (69.7%)	21 (75.0%)	
Engraftment syndrome ^+,a^			0.59			0.68
Yes	14 (22.2%)	3 (30.0%)		10 (21.7%)	7 (25.9%)	
No	49 (77.8%)	7 (70.0%)		36 (78.3%)	20 (74.1%)	
Hospitalization duration ^b^	13 (12–14)	13 (13–16)	0.21	13 (12–14)	13 (12–16)	0.20
Hospital re-admission within 84 days ^a^	7 (7.4%)	7 (30.4%)	0.002	5 (7.2%)	9 (18.8%)	0.06
Death within 84 days ^a^	0 (0.0%)	0 (0.0%)	ND	0 (0.0%)	0 (0.0%)	ND

Shown are melphalan total dose; CD34+ cell dose infused; times to achieving ANC and platelet engraftments; total length of hospital stay; *Enterobacterales* BSI for participants for whom blood cultures were obtained; and numbers of participants with re-admission or death before day 84 after transplantation for participants classified by FRE colonization on pre-transplant and any of the three time points sampled. PBSC = peripheral blood stem cells, FRE = fluoroquinolone-resistant *Enterobacterales*, ABW = actual body weight, ANC = absolute neutrophil count, RBC = red blood cell, ICU = intensive care unit. ^a,b,c^ Data are shown as (a) n (%), (b) median (IQR), or (c) mean (SD). See Methods for a description of statistical analysis. ^#^ Shown are the numbers of participants receiving either ciprofloxacin or levofloxacin prophylaxis as described in the Methods. * Shown are results of initial blood cultures obtained for participants with febrile neutropenia or as otherwise ordered by physicians for participants without febrile neutropenia. ^+^ Engraftment syndrome analysis limited to subjects with unplanned corticosteroid use. Participants with planned corticosteroid use not included in statistical analysis.

**Table 5:** Sequencing of BSI and peri-anal swab isolates.

Subject Study No.	Pre-Transplant	Hospital Discharge	Day 100	BSI
FRE-050	*Klebsiella* *	Negative	*E. coli* and *Klebsiella*	*Klebsiella* *
FRE-056	*E coli* and *Klebsiella*	ND ^+^	ND ^+^	*E coli*
FRE-105	*E coli* *	Negative	Negative	*E coli* *

Shown are the organisms detected on peri-anal swabs and blood cultures for the three subjects who developed a Gram-negative BSI. * These organisms are identical based on whole-genome sequencing. ^+^ Peri-anal swab not obtained at this time point.

**Table 6:** Re-hospitalization after initial discharge.

Subject Study No.	FRE Colonized	Day Re-Admit	Admission Diagnosis	Fever	BSI *
FRE-038	Yes	32	Atrial fibrillation	No	ND
FRE-039	Yes	17	Fever with abdominal pain	Yes	Neg
FRE-043	Yes	16	Fever from engraftment syndrome	Yes	Neg
FRE-056	Yes	29	Diarrhea–Salmonella	Yes	Neg
FRE-057	No	28	SARS-CoV-2	Yes	Neg
FRE-069	No	18	Coronary artery disease with bypass grafting	No	ND
FRE-076	No	57	Mood disorder	No	ND
FRE-087	Yes	73	Thrombocytopenia	No	ND
FRE-089	Yes	26	Cellulitis of foot	No	Neg
FRE-092	No	25	Diarrhea-Salmonella	No	ND
FRE-099	Yes	21 35 53	Hypotension SARS-CoV-2 Fever	No Yes Yes	ND Neg Neg
FRE-102	No	23	Fever	Yes	Neg
FRE-103	Yes	24 41	Urinary infection SARS-CoV-2	Yes Yes	Neg Neg
MFRE-006	Yes	Unk	Urinary infection	Unk	Unk

Shown are the subjects re-admitted to the inpatient unit by day of re-admission after transplantation. Also shown are the primary reasons for re-admission, if fever was reported, and if bloodstream infection was reported. FRE = fluoroquinolone-resistant *Enterobacterales*, SARS-CoV-2 = severe acute respiratory syndrome coronavirus 2. Subject number preceded by “M” underwent transplantation at MGUH. * BSI = bloodstream infection detected on blood culture, Neg = no growth in blood cultures, ND is blood culture not performed, Unk = unknown.

## Data Availability

De-identified subject data will be made available on request after publication of this manuscript. Investigational Review Board (IRB) approval and data sharing agreement for investigational use of these data may be required for receipt of these data files. Please submit your request for access to the de-identified data set to Christine Cho, MD, Christine.cho@hmhn.org.

## Supplementary Materials

The following supporting information can be downloaded at: https://www.mdpi.com/article/10.3390/cancers18101566/s1: Supplementary Table S1: Subjects and results of testing for FRE colonization (including pre-apheresis testing for subjects receiving chemotherapy mobilization of PBSCs not included in primary or secondary analyses).

## Abbreviations

ABW	Actual body weight
ANC	Absolute neutrophil count
aSCT	Autologous stem cell transplantation
BSI	Bloodstream infection
BSA	Body surface area
CD34+	Cluster of differentiation 34-positive
CDE	Cyclophosphamide, dexamethasone and etoposide
CLSI	Clinical and Laboratory Standard Institute
CMV	Cytomegalovirus
COVID-19	Coronavirus disease 2019
ESBL	Extended-spectrum B-lactamase
ESBL-E	Extended-spectrum B-lactamase-producing Enterobacterales
FN	Febrile neutropenia
FQ	Fluoroquinolone
FRE	Fluoroquinolone-resistant Enterobacterales
G-CSF	Granulocyte colony-stimulating factor
GI	Gastrointestinal
HUMC	Hackensack University Medical Center
ICU	Intensive care unit
IgA	Immunoglobulin A
IgG	Immunoglobulin G
IQR	Interquartile range
IV	Intravenous
Kg	Kilogram
m^2^	Square meters
MGUH	MedStar Georgetown University Hospital
MRSA	Methicillin-resistant Streptococcus aureus
NA	Not available
Neg	Negative
ND	Not determined
PBSC	Peripheral blood stem cell
PICC	Peripherally inserted central catheter
Pos	Positive
QC	Quality control
RBC	Red blood cell
R-ISS	Revised International Staging System
SC	Subcutaneous
SD	Standard deviation
SNV	Single-nucleotide variant
SARS-CoV-2	Severe acute respiratory syndrome coronavirus 2
TPO	Thrombopoietin
Unk	Unknown
WGS	Whole-genome sequencing
