# Supplementary material for: Fluoroquinolone Prophylaxis Uncovers High Prevalence Rates of Fluoroquinolone-Resistant Enterobacterales Colonization in Multiple Myeloma Autologous Transplant Patients: A Prospective Cohort Study
Source: Cancers (Basel). 2026 May 12;18(10):1566. doi: 10.3390/cancers18101566 (PMC13204931; doi:10.3390/cancers18101566)
Supplement: Supplementary file 1 [file cancers-18-01566-s001.zip › cancers-4282388-supplementary.pdf]

SUPPLEMENTAL MATERIAL

SUPPLEMENTAL TABLES

Table S1: Subjects and Results of Testing for FRE Colonization

	Pre-Apheresis		Pre-Transplant		Hospital Discharge		Day 100	
	**Result**	**Organism**	**Result**	**Organism**	**Result**	**Organism**	**Result**	**Organism**
FRE-001	ND		Pos	E. coli	Pos	E. coli<br>Kleb sp	Neg	
FRE-002	Neg		Neg		Neg		Neg	
FRE-003	Pos	E. coli	Neg		Pos	Kleb sp*	Pos	E. coli*
FRE-004	Neg		Neg		Neg		Pos	Kleb sp*
FRE-005	Neg		Neg		Neg		Neg	
FRE-006	Neg		Neg		Neg		Pos	E. coli
FRE-007	Neg		Neg		Neg		Pos	Kleb sp*
FRE-008	ND		Neg		Pos	Kleb sp*	ND	
FRE-009	Pos	Kleb sp*	Neg		Pos	Kleb sp*	Neg	
FRE-010	ND		Pos	E. coli*	Pos	E. coli*	Pos	E. coli
FRE-011	Neg		Neg		Pos	Kleb sp*	Neg	
FRE-012	Neg		Neg		Neg		Pos	Kleb sp*
FRE-013	Neg		Neg		Neg		Neg	
FRE-014	NA		Neg		Neg		Neg	
FRE-016	Neg		Neg		Neg		Neg	
FRE-017	ND		Neg		Neg		Neg	
FRE-018	Neg		Pos	E. coli*	Pos	E. coli*	Neg	
FRE-019	ND		Neg		Neg		Pos	E. coli*
FRE-020	ND		Neg		ND		Neg	
FRE-021	Pos	E. coli	Neg		Pos	E. coli*	Neg	
FRE-022	ND		Neg		ND		Neg	
FRE-023	ND		Neg		Neg		Neg	
FRE-024	Pos	E. coli	Neg		Pos	E. coli	Pos	E. coli
FRE-025	Neg		Neg		Neg		ND	
FRE-026	ND		Neg		Neg		Neg	
FRE-027	Neg		Neg		Neg		Neg	
FRE-028	Neg		Neg		Neg		Neg	
FRE-029	ND		Neg		Fail		Neg	
FRE-030	ND		Neg		ND		ND	
FRE-031	Pos	E. coli	Pos	E. coli	Pos	E. coli	Pos	E. coli
FRE-032	Neg		Neg		Pos	Kleb sp*	ND	
FRE-033	ND		Neg		Neg		Neg	
FRE-034	ND		Pos	Kleb sp*	Pos	E coli<br>Kleb sp*	Pos	E coli*<br>Kleb sp*
FRE-035	ND		Neg		Neg		Neg	
FRE-036	ND		Pos	E. coli	Pos	E. coli	Pos	E. coli
FRE-037	ND		Pos	E. coli*<br>Kleb sp*	Pos	E. coli<br>Kleb sp*	Pos	E. coli<br>Kleb sp*
FRE-038	ND		Pos	E. coli	Neg		Pos	Kleb sp*
FRE-039	Neg		Pos	Kleb sp*	Neg		Pos	E. coli*
FRE-042	ND		Neg		Neg		Neg	
FRE-043	ND		Neg		Pos	E. coli	Pos	E. coli
FRE-044	Pos	Kleb sp*	Pos	E. coli*	Pos	E. coli*	Pos	Kleb sp*
FRE-045	Pos	E. coli*	Pos	E. coli*	Pos	E. coli*	Neg	
FRE-046	Neg		Neg		Neg		Neg	
FRE-047	ND		Neg		Neg		ND	
FRE-048	Pos	E. coli<br>Kleb sp	Neg		Neg		Neg	
FRE-049	Neg		Neg		Neg		Neg	

FRE-050	Pos	Kleb sp	Pos	Kleb sp	Neg		Pos	E. coli*<br>Kleb sp*
FRE-051	ND		Neg		Pos	E. coli	ND	
FRE-052	Neg		Neg		Neg		Neg	
FRE-053	Neg		Neg		Neg		Neg	
FRE-054	Neg		Neg		Neg		Neg	
FRE-055	ND		Neg		Neg		Neg	
FRE-056	ND		Pos	E. coli*<br>Kleb sp*	ND		ND	
FRE-057	Neg		Neg		Neg		Neg	
FRE-058	Neg		Neg		Neg		Neg	
FRE-059	ND		Neg		Neg		Neg	
FRE-060	ND		Neg		Neg		Neg	
FRE-061	ND		Neg		Neg		Neg	
FRE-062	Neg		Neg		Neg		Neg	
FRE-063	Neg		Neg		Neg		Neg	
FRE-064	ND		Neg		Neg		Neg	
FRE-066	Neg		Neg		ND		ND	
FRE-067	ND		Neg		Neg		Neg	
FRE-068	Neg		Neg		Neg		Pos	Kleb sp*
FRE-069	ND		Neg		Neg		Neg	
FRE-070	Neg		Neg		Neg		Neg	
FRE-071	ND		Neg		Neg		Neg	
FRE-072	Neg		Neg		ND		ND	
FRE-073	ND		Neg		Neg		Pos	Kleb sp
FRE-074	ND		Neg		Neg		Neg	
FRE-075	Neg		Neg		Neg		Neg	
FRE-076	Neg		Neg		Neg		Neg	
FRE-077	Neg		Neg		Neg		Neg	
FRE-078	Neg		Neg		Neg		Neg	
FRE-079	ND		Neg		Neg		Neg	
FRE-080	ND		Neg		Pos	Kleb sp*	Pos	Kleb sp*
FRE-081	ND		Neg		Neg		Neg	
FRE-082	ND		Neg		Pos	Kleb sp*	Neg	
FRE-083	Neg		Neg		Pos	Kleb sp*	Neg	
FRE-084	Pos	E. coli	Pos	E. coli	Pos	E. coli	Neg	
FRE-085	ND		Neg		Neg		Neg	
FRE-086	Neg		Neg		Neg		Pos	Kleb sp*
FRE-087	ND		Pos	Kleb sp	Pos	E. coli*<br>Kleb sp*	Pos	E. coli*
FRE-088	ND		Pos	E. coli	Pos	E. coli*	Pos	E. coli
FRE-089	ND		Pos	E. coli	Pos	E. coli	Pos	Kleb sp*
FRE-090	ND		Neg		Neg		Pos	E. coli*
FRE-091	ND		Neg		Neg		Pos	Kleb sp*
FRE-092	ND		Neg		Neg		ND	
FRE-093	ND		Neg		Neg		ND	
FRE-094	ND		Neg		Neg		Neg	
FRE-095	Neg		Neg		Neg		Neg	
FRE-096	ND		Neg		Neg		Fail	
FRE-097	ND		Neg		Pos	Kleb sp*	ND	
FRE-098	ND		Neg		ND		ND	
FRE-099	ND		Neg		Pos	Kleb sp*	Pos	E. coli*
FRE-100	ND		Neg		ND		ND	
FRE-101	ND		Pos	Kleb sp*	Neg		Neg	
FRE-102	ND		Neg		Neg		Fail	
FRE-103	ND		Pos	Kleb sp*	Neg		Pos	E. coli*
FRE-104	ND		Neg		ND		ND	
FRE-105	ND		Pos	E. coli*	Neg		Neg	

MFRE-001	ND		Neg		Pos	Kleb sp*	ND	
MFRE-003	ND		Neg		Neg		Neg	
MFRE-004	ND		Neg		Neg		Neg	
MFRE-005	ND		Neg		Neg		Neg	
MFRE-006	ND		Pos	E. coli	ND		ND	
MFRE-007	ND		Neg		ND		ND	
MFRE-008	ND		Neg		ND		ND	
MFRE-009	ND		Neg		ND		ND	
MFRE-010	ND		Neg		ND		ND	
MFRE-011	ND		Neg		Neg		Neg	
MFRE-013	ND		Pos	E. coli	Pos	E. coli	ND	
MFRE-015	ND		Neg		Neg		ND	
MFRE-016	Neg		Neg		ND		ND	
MFRE-017	ND		Neg		ND		Neg	
MFRE-018	Pos	E. coli*	Pos	E. coli*<br>Kleb sp*	ND		ND	
MFRE-019	ND		Neg		ND		Neg	

Shown are the results of all samples by timing of test. The pre-apheresis sample is shown for completeness of the dataset but the data at this time point were not used in primary or secondary analyses. Subjects are listed by study number and site (study number preceded by "M" underwent transplantation at MGUH). ND=Sample not obtained if PBSC collected with G-CSF/plerixafor (pre-apheresis sample) or if subject declined to collect sample, Neg=FRE not identified, Pos=FRE identified in sample, Fail=Positive control failed. E.coli=*Escherichia coli*, Kleb sp=*Klebsiella* species.

*Designates ESBL-producing organism.
